# Association of the C47T Polymorphism in SOD2 with Amnestic Mild Cognitive Impairment and Alzheimer's Disease in Carriers of the APOE*ε*4 Allele

**DOI:** 10.1155/2015/746329

**Published:** 2015-12-01

**Authors:** David Gamarra, Xabier Elcoroaristizabal, Manuel Fernández-Martínez, Marian M. de Pancorbo

**Affiliations:** ^1^BIOMICS Research Group, Department of Zoology and Cellular Biology A, Lascaray Research Center (CIEA), University of the Basque Country (UPV/EHU), Vitoria-Gasteiz, 01006 Alava, Spain; ^2^Department of Neurology, Hospital Universitario Cruces, BioCruces Health Research Institute, Barakaldo, 48093 Bizkaia, Spain

## Abstract

Oxidative stress plays an important part in amnestic mild cognitive impairment (aMCI), the prodromal phase of Alzheimer's disease (AD). Recent evidence shows that polymorphisms in the* SOD2* gene affect the elimination of the reactive oxygen species (ROS) generated in mitochondria. The aim of this study was to determine whether the functional rs4880 SNP in the* SOD2* gene is a risk factor associated with aMCI and sporadic AD. 216 subjects with aMCI, 355 with AD, and 245 controls have been studied. The SNP rs4880 of the* SOD2* gene was genotyped by RT-PCR and the* APOE* genotype was determined by PCR and RFLPs. Different multinomial logistic regression models were used to determine the risk levels for aMCI and AD. Although the T allele of the SOD2 rs4880 SNP gene (rs4880-T) is not an independent risk for aMCI or AD, this allele increases the risk to aMCI patients carrying at least one APOE*ε*4 allele. Moreover, rs4880-T allele and APOE*ε*4 allele combination has been found to produce an increased risk for AD compared to aMCI reference patients. These results suggest that APOE*ε*4 and rs4880-T genotype may be a risk for aMCI and a predictor of progression from aMCI to AD.

## 1. Introduction

Neuropathologically, the accumulation of beta-amyloid (A*β*) and tau proteins in the brain tissue of patients with Alzheimer's disease (AD) is related to the loss of synapse and neuronal death. The increase in oxidative stress seems to be one of the possible causes of the aetiology of AD, probably due to the loss of physiological control of the reactive oxygen species (ROS) [1]. Animal models have allowed correlating the increase in oxidative stress to the increase in the levels and plaques of A*β* and oxidative damage [2]. Moreover, a prodromal phase of AD, amnestic mild cognitive impairment (aMCI), has been accepted as a transition phase between normal aging and Alzheimer's disease (AD) [3, 4]. In fact, aMCI patients have shown a greater risk for developing AD and convert at an annual rate of 10% to 15%, compared with 1%-2% in the general elderly population [5, 6]. The brains of patients with AD in its prodromal phase, with amnesiac mild cognitive impairment (aMCI), have shown significant oxidative damage [7–10] and it is suggested that this damage affects the nuclear DNA differently from the mitochondrial one, the latter being greater [11]. Peripheral oxidative stress biomarkers in AD patients have showed an increase in molecules such as carbonyl proteins [12], 3-nitrotyrosine [13], isoprostanes [14], DNA oxidation (8-oxoguanine) [15], and malonaldehyde (MDA) [16]. Furthermore, higher MDA and carbonyl protein levels have been found in MCI [16, 17] reporting a clear evidence of oxidative damage in mild cognitive impairment too.

Mitochondria generate a significant amount of ROS in normal activity. These are eliminated by antioxidant enzymes, such as manganese superoxide dismutase (MnSOD) [18, 19]. Animal models have shown that there is a compensatory induction of MnSOD in response to an initial increase in oxidative stress, which protects neurons from A*β* toxicity [20, 21]. Nevertheless, continued exposure to oxidative damage can suppress expression of MnSOD causing cell death in mature neurons [22]. This may be related to insufficient protection to oxidative damage in the brain [23, 24]. Additionally, MnSOD activity diminishes with age, in keeping with the progression from aMCI to AD, leading to an increase in ROS and exacerbating the pathogenesis related to AD [25–27].

The* SOD2* gene (6q25) encodes the MnSOD and has several single nucleotide polymorphisms (SNPs), among which rs4880 (C/T) is included (also designated as C47T, Ala16Val, Ala-9Val or A16V). The T allele of this SNP (rs4880-T) has been associated with changes in the activity of MnSOD as a result of modification to the mitochondrial targeting sequence (MTS) [18, 21, 28], which may play a part in neurodegenerative processes. The results of the studies of this polymorphism associated with neurodegenerative illnesses are diverse, probably due to the different involvement of the antioxidant enzymes in these illnesses [29–38]. Specifically, the rs4880-T allele has been associated with a greater risk of familial AD [39], while not seeming to change the risk of sporadic AD [40]. Nevertheless, to date, no study has evaluated the rs4880 SNP in aMCI patients.

Apolipoprotein E (apoE), the main susceptibility factor for aMCI and AD [41, 42], also has antioxidant properties that vary depending on its isoforms E2 > E3 > E4 [43]. Additionally, there is a relationship between these apoE isoforms and certain antioxidant enzymes. Thus, in brain tissue from AD patients, the activities of catalase and glutathione peroxidase in carriers of the E4 isoform are reduced [44]. Moreover, AD patients with APOE4 had shown higher blood hydroxyl radical levels than those without this allele or nondemented subjects [45]. This suggests that this isoform interacts with the antioxidant systems, at least the cytoplasmic ones. The E4 isoform has also been related to mitochondrial dysfunction and neurotoxicity [46, 47]. Nevertheless, the molecular mechanism responsible for the antioxidant capacity of apoE is still unknown [48].

The* SOD2* SNP rs4880 and the* APOE* genotypes are related to the cell antioxidant activity. The sum of specific alleles in both genes could confer greater susceptibility to the development of AD from its prodromal stage, aMCI. The aim of this study was to find the effect of the presence of the T allele of* SOD2* SNP rs4880 (rs4880-T) in combination with the APOE*ε*4 allele on the risk of aMCI and AD. In order to do this, we have carried out the genotyping of* APOE* and* SOD2* in a case-control study.

## 2. Materials and Methods

### 2.1. Subjects

816 samples were collected at neurology services of various hospitals in the Basque Country and included in National DNA Bank (Genome Foundation Spain). Patients with aMCI were diagnosed according to the Petersen criteria. Patients considered to have aMCI should show impaired memory and any reduction in their functions should be confirmed by an informant, with less than 0.5 on the CDR scale and normal state in the performance of other cognitive functions and routine activities. The figures obtained were adjusted for age and level of education. The diagnosis of AD was based on the DSM IV and the NINCDS-ADRDA criteria for AD. Patients with a total score of less than 3 (1 and 2) on the CDR scale (mild to moderate dementia) were included. Healthy control subjects had to obtain a score of 0 CDR within the normal range for their age and level of education in the psychometric tests. According to the test results, the participants were classified into the following groups: patients with aMCI (*n* = 216), patients with AD (*n* = 355), and healthy control subjects (*n* = 245). Additionally, the biochemical and neuroimaging criteria published in Martínez et al. (2009) were taken into account [49].

This study was approved by the Ethics Committee of Cruces Hospital (Barakaldo, Biscay) and was carried out in accordance with the Declaration of Helsinki on biomedical research involving human beings. Informed consent was obtained before the start of the genetic and clinical tests.

### 2.2. Genetic Analysis

Samples of peripheral blood were taken from all the individuals using Vacutainer tubes with EDTA anticoagulant. The DNA was extracted by proteolytic lysis and purified using phenol/chloroform followed by ethanol precipitation. Genetic analyses were carried out without prior knowledge of the diagnosis (aMCI, AD, and healthy controls). The rs4880 SNP was genotyped using a TaqMan allelic discrimination assay (AB C_8709053_10) on an ABI PRISM 7000 SDS. The thermocycling conditions were as follows: 95°C 10 min, 40 cycles 95°C 15 sec, and 58°C 1 min 30 sec.* APOE* was amplified with the 112F and 158R primers under the conditions described by Wilton and Lim [50]. The genotype was obtained by digestion of the PCR product with* Hae II* and* Afl III* restriction enzymes under the conditions described by Alvarez-Alvarez et al. [51].

### 2.3. Statistical Analysis

The one-way ANOVA test was used to observe the differences in the demographic variables of age and the MMSE scores. Levene test was applied for homoscedasticity test and Dunnett's T3 test was computed. The goodness of fit for the Hardy-Weinberg equilibrium was estimated using the exact test in Guo and Thompson (1992) [52] using the Genepop v4.0 program. The *G*-test was used to check the differences of allelic and genotypic distributions between the groups of patients and controls with Bonferroni correction. Power for Genetic Association Analyses (PGA) package [53] has been used to compute the power of this case-control study.

Diverse logistic regression models were examined using the SPSS v22.0 software. Models were run under the assumption of additive (AA versus Aa versus aa), dominant (AA versus Aa/aa), or recessive (AA/Aa versus aa) inheritance in aMCI and AD. First, the risk of APOE*ε*4 allele was evaluated. Secondly, rs4880-T allele and rs4880 genotypes risks were evaluated. Finally, rs4880-T and APOE*ε*4 interaction terms to test for epistatic effects were computed. Control subjects adjusted for age and sex were used as a reference category to evaluate AD and aMCI risk. In addition, aMCI was also used as a reference category to evaluate AD risk. The *p* < 0.05 values were considered significant.

## 3. Results

The MMSE scores in the aMCI, AD, and control groups were 25.93 ± 2.38, 18.84 ± 5.01, and 28.05 ± 1.56, respectively. The comparison of averages in the MMSE showed statistically significant differences as expected.

Table 1 shows the age distribution and proportion of sexes in the three groups studied. The proportion of women was higher in all cases, with no statistically significant differences between aMCI and controls. In terms of the average age, no statistically significant differences were shown.

The allelic and genotypic frequencies of the* SOD2* rs4880 SNP and the* APOE* gene of each groups studied are shown in Table 2. The T risk allele frequency of the rs4880 SNP was 0.488 for aMCI and 0.463 for AD, being similar to the frequency observed in the controls (0.480). Therefore, significant differences between the aMCI, AD, and healthy control groups were not found in rs4880 polymorphism (*p* > 0.05). AD cases and controls fit the Hardy-Weinberg equilibrium (HWE) but not the aMCI group. A statistically significant deviation was found in aMCI cases due to a deficiency of CC homozygotes with a frequency of 0.218 being the frequency value expected of 0.259.

APOE*ε*4 allele frequency was 0.250 in aMCI and 0.298 in AD, being higher than frequency in controls (0.104). Controls did not show any *ε*4*ε*4 genotype while a higher frequency in *ε*3*ε*4 and *ε*4*ε*4 genotypes was found in aMCI (0.305 and 0.093, resp.) and AD (0.428 and 0.076, resp.). These differences in* APOE* allelic and genotypic frequencies appeared to be statistically significant (*p* < 0.001) between aMCI versus controls and AD versus controls, although not between aMCI versus AD.

Multiple multinomial logistic regression analyses were carried out to check whether the rs4880 allele is a risk factor for aMCI and AD using different models as shown in Table 3 (additive, CT versus CC and TT versus CC; dominant, CT/TT versus CC; and recessive, TT versus CC/CT). The odds ratio (OR) obtained was not significant in any of the models. None of the genotypes (TT and TC) of the rs4880 SNP showed statistical significance, unlike the APOE*ε*4 allele which was statistically significant (Table 3).

Subsequently, the epistatic effect of the rs4880-T and the APOE*ε*4 alleles was evaluated (Table 3). To this end, the OR in carriers of the APOE*ε*4 allele and at least one rs4880-T allele was calculated and the results obtained were statistically significant. The risk for aMCI patients was nominally increased in carriers of APOE*ε*4 allele combined with rs4880-T allele in the dominant model (OR = 3.17; 95% CI = 1.80–5.84, *p* < 0.001). Moreover, aMCI patients also showed an increased risk in carriers of the APOE*ε*4 allele combined with TC genotype in the additive model (OR = 3.54; 95% CI = 1.82–6.86, *p* < 0.001). However, the epistatic effects of the rs4880-T and the APOE*ε*4 alleles have not shown an increased risk for Alzheimer's disease in comparison with results obtained considering only the APOE*ε*4 allele under different models and control patients as the reference category. On the other hand, when aMCI patients were used as the reference category, a significant risk for AD was observed in patients with APOE*ε*4 allele (OR = 1.64; 95% CI = 1.16–2.32, *p* < 0.001) and also an epistatic effect was observed for AD in carriers of the APOE*ε*4 allele and the rs4880-TT genotype in the recessive model (OR = 2.05; 95% CI = 1.09–3.87, *p* < 0.001) (Supplemental Table in Supplementary Material available online at http://dx.doi.org/10.1155/2015/746329).

## 4. Discussion

Oxidative stress is an age-related process that increases the risk of developing neurodegenerative illnesses. Various studies show the involvement of oxidative stress and mitochondrial dysfunction in neuronal injury and death, both during the initial stages of aMCI, and once AD has developed [54–57]. For this reason, the cellular processes involved in the detoxification of ROS have been considered of relevance to research into the factors of genetic susceptibility that promote cognitive impairment.

In our study, we have seen that the T allele of the* SOD2* rs4880 SNP (rs4880-T) is more common in the aMCI group than in the healthy control group. Moreover, the aMCI group in particular does not comply with the Hardy-Weinberg equilibrium for this SNP, which seems to indicate a possible association between the rs4880-T allele and the disease. However, the absence of significant risk in the multinomial logistic regression models suggests that the rs4880-T allele is not a risk factor for aMCI and AD. These results coincide with those of Ventriglia et al. [40] in AD patients, but it has not been possible to contrast them with aMCI due to the absence of prior studies.

Both* SOD2* and* APOE* encode proteins with an antioxidant capacity [18, 41]. For this reason, despite the lack of risk conferred by the rs4880-T allele, it is important to evaluate its interaction with the APOE*ε*4 allele, because the combination of both alleles may increase the individual effects [58]. The instability of the mRNA product of the rs4880-T allele and the deficient amount to the mitochondrial matrix of the encoding isoform can negatively affect the formation of active SOD2 tetramer and, therefore, contribute to the increase of oxidative stress [59, 60]. On the other hand, the rs4880-C allele allows a more efficient import to the mitochondrial matrix to form 40% more active tetramer than the rs4880-T allele [60].

Our results showed a slight nominal increase in the risk of only aMCI in carriers of the rs4880-T and APOE*ε*4 alleles. Therefore, the results seem to indicate that the rs4880-T allele may be implicated in the increased risk of developing aMCI. This could be due to oxidative stress as an important factor in aMCI development but other neurodegenerative factors could also be responsible in the progression of healthy individuals to Alzheimer's disease. The strengths of our work are its multicentre nature and the inclusion of patients with aMCI, AD, and healthy controls in the same study to investigate the association of the rs4880 SNP of the* SOD2* gene for the first time, in both AD and aMCI, the stage prior to AD. Thus, it has been possible to see that the risk of the combination of the rs4880-T and APOE*ε*4 alleles (OR = 3.17) has been calculated with sufficient statistical power (90.9%). An increased risk (OR = 3.54) with sufficient statistical power (99.9%) has also been observed in rs4880-TC genotype and carriers of APOE*ε*4 for *α* = 0.05. However, the risk of the rs4880-TT and APOE*ε*4 combination could not be evaluated due to lack of statistical power (74.5%).

MCI has become a deal of interest, partly because the identification of patients at an early stage of AD may enable the initiation of treatment strategies when they are most likely to be effective. Despite the numerous genetic studies of AD, the influence of genetic variation on progression from MCI to AD has been poorly studied. Although MCI patients have shown an increased risk for Alzheimer's disease (AD) [61], it is known that APOE*ε*4 is the strongest genetic risk for AD. Further analysis has revealed that MCI patients with an APOE*ε*4 allele have twice (-/*ε*4) and four times (*ε*4/*ε*4) higher risk to convert to AD than those without an APOE*ε*4 allele [62], whereas annual conversion rate to AD seems to be increased from 20%, in non-APOE*ε*4 carriers, to 32.5% in patients with APOE*ε*4 [63]. Therefore, the interaction of APOE*ε*4 with other genes may possibly increase the prognostic accuracy. Although we could not calculate conversion rates due to an absence in monitoring of patients, we considered risk for AD patients with APOE*ε*4 and rs4880-T compared with an aMCI reference group. We found a significant risk for AD in patients with APOE*ε*4 allele (OR = 1.56) compared with non-APOE*ε*4 carriers. Moreover, an increased risk for AD in patients with APOE*ε*4 and rs4880-TT genotype (OR = 2.05) was found (statistical power > 80%), suggesting that an epistatic effect in those polymorphisms could increase risk to develop AD from aMCI patients.

In conclusion, the rs4880-T allele of the* SOD2* gene is not an independent risk factor for aMCI and AD patients, although this allele in combination with the APOE*ε*4 allele produces an increase in the risk for aMCI. Finally, rs4880-T allele and APOE*ε*4 allele combination has also been found to produce an increased risk for AD compared to aMCI patients. These data need to be confirmed in further studies, and as new susceptibility variant has been identified, it needs to be confirmed in more MCI studies taking into account time-dependent progression to AD.

## Supplementary Material

Patients with aMCI were used as the reference category to compute multinomial logistic regression models of rs4880-T (SOD2) and APOEɛ4 allele in AD. As a result, an epistatic effect was found in AD patients in contrast to aMCI patients (Recessive model).

## Figures and Tables

**Table 1:** Demographic characteristics.

Group	*n* (Men/women)	Age^a^
aMCI	216 (84/132)	72.02 ± 7.88
AD	355 (103/252)	75.03 ± 7.70
Controls	245 (101/144)	74.81 ± 10.23

^a^Age, mean ± standard deviation (SD).

**Table 2:** Allelic and genotypic frequencies.

Gene			aMCI (*N* = 216)	AD (*N* = 355)	Controls (*N* = 245)
*SOD2*	Allele	T	0.488	0.463	0.480
		C	0.512	0.537	0.520
	Genotype	TT	0.194	0.220	0.221
		TC	0.588	0.487	0.518
		CC	0.218	0.293	0.261
	H-W^a^	*p*-value^b^	0.012	0.755	0.608

*APOE*	Allele	2	0.025	0.034	0.057
		3	0.725	0.668	0.839
		4	0.250	0.298	0.104
	Genotype	2/2	0.000	0.000	0.009
		2/3	0.042	0.050	0.090
		2/4	0.009	0.018	0.008
		3/3	0.551	0.428	0.693
		3/4	0.305	0.428	0.200
		4/4	0.093	0.076	0.000
	H-W^a^	*p*-value^b^	0.115	0.567	0.087

^a^Hardy-Weinberg equilibrium. ^b^Significative *p* value < 0.025 after Bonferroni correction.

**Table 3:** Multinomial logistic regression models of rs4880-T (*SOD2*) and APOE*ε*4 allele in aMCI and AD patients.

Gene		Model	aMCI	*p*	AD	*p*
			OR CI 95%		OR CI 95%	
*APOE*		*ε*4 (+)	2.54 (1.68–3.83)	<0.001	4.15 (2.85–6.05)	<0.001

*SOD2*	Additive	TT	1.03 (0.59–1.80)	0.914	0.91 (0.57–1.46)	0.708
	Additive	TC	1.34 (0.85–2.11)	0.206	0.85 (0.57–1.25)	0.415
	Dominant	TC/TT	1.25 (0.81–1.93)	0.318	0.87 (0.60–1.26)	0.455
	Recessive	TT	0.84 (0.53–1.33)	0.455	1.01 (0.68–1.50)	0.943

*APOE∗SOD*2	Additive	*ε*4 (+) *∗* TC	3.54 (1.82–6.86)	<0.001	3.48 (1.94–6.27)	<0.001
	Additive	*ε*4 (+) *∗* TT	2.39 (1.00–5.74)	0.051	3.28 (1.54–6.99)	0.002
	Dominant	*ε*4 (+) *∗* TC/TT	3.17 (1.80–5.84)	<0.001	3.41 (2.01–5.80)	<0.001
	Recessive	*ε*4 (+) *∗* TT	1.95 (0.89–4.27)	0.095	4.00 (2.00–7.99)	0.002

Control subjects were the reference category in all models. OR, odds ratio. CI, confidence interval.
