# Supplementary material for: Association of the C47T Polymorphism in SOD2 with Amnestic Mild Cognitive Impairment and Alzheimer's Disease in Carriers of the APOEε4 Allele
Source: Dis Markers. 2015 Dec 1;2015:746329. doi: 10.1155/2015/746329 (PMC4678069; doi:10.1155/2015/746329)
Supplement: Supplementary file 1 — Patients with aMCI were used as the reference category to compute multinomial logistic regression models of rs4880-T (SOD2) and APOEɛ4 allele in AD. As a result, an epistatic effect was found in AD patients in contrast to aMCI patients (Recessive model). [file 746329.f1.docx]

**Supplemental table. Multinomial logistic regression models of rs4880-T (*SOD2*) and APOEε4 allele in AD patients using aMCI patients as a reference category.**

| **Gene** |  | **Model** | **OR CI95%** | ***p*** |
| --- | --- | --- | --- | --- |
| ***APOE*** |  | **ε4 (+)** | 1.64 (1.16–2.32) | 0.005 |
| ***SOD2*** | **Additive** | **TT** | 0.88 (0.53-1.48) | 0.647 |
|  | **Additive** | **TC** | 0.63 (0.42-0.96) | 0.034 |
|  | **Dominant** | **TC/TT** | 0.70 (0.46-1.04) | 0.777 |
|  | **Recesive** | **TT** | 1.21 (0.79-1.85) | 0.385 |
| ***APOE*SOD2*** | **Additive** | **ε4(+)* TT** | 1.37 (0.65-2.87) | 0.403 |
|  | **Additive** | **ε4(+)* TC** | 0.98 (0.55-1.75) | 0.956 |
|  | **Dominant** | **ε4(+)* TC/TT** | 1.08 (0.62-1.86) | 0.790 |
|  | **Recesive** | **ε4(+)* TT** | 2.05 (1.09-3.87) | 0.026 |

OR, Odd Ratio. CI, conﬁdence interval
